# Psoriasiform Inflammation Is Associated with Mitochondrial Fission/GDAP1L1 Signaling in Macrophages

**DOI:** 10.3390/ijms221910410

**Published:** 2021-09-27

**Authors:** Ahmed Alalaiwe, Chi-Yuan Chen, Zi-Yu Chang, Jui-Tai Sung, Shih-Yi Chuang, Jia-You Fang

**Affiliations:** 1 Department of Pharmaceutics, College of Pharmacy, Prince Sattam Bin Abdulaziz University, Al Kharj 11942, Saudi Arabia; alalaiwe@gmail.com; 2 Graduate Institute of Health Industry Technology, Chang Gung University of Science and Technology, Kweishan, Taoyuan 333, Taiwan; d49417002@gmail.com; 3 Research Center for Food and Cosmetic Safety and Research Center for Chinese Herbal Medicine, Chang Gung University of Science and Technology, Kweishan, Taoyuan 333, Taiwan; 4 Tissue Bank, Chang Gung Memorial Hospital, Kweishan, Taoyuan 333, Taiwan; 5 Department of Traditional Chinese Medicine, Chang Gung Memorial Hospital, Keelung 204, Taiwan; changzhi887@gmail.com; 6 Institute of Traditional Medicine, School of Medicine, National Yang Ming Chiao Tung University, Taipei 112, Taiwan; 7 Pharmaceutics Laboratory, Graduate Institute of Natural Products, Chang Gung University, Kweishan, Taoyuan 333, Taiwan; fajy5521@gmail.com; 8 Department of Anesthesiology, Chang Gung Memorial Hospital, Kweishan, Taoyuan 333, Taiwan

**Keywords:** psoriasis, macrophage, mitochondrial fission, GDAP1L1, Drp1, imiquimod

## Abstract

While psoriasis is known as a T cell- and dendritic cell-driven skin inflammation disease, macrophages are also reported to play some roles in its development. However, the signaling pathway of activated macrophages contributing to psoriasis is not entirely understood. Thus, we aimed to explore the possible mechanisms of how macrophages initiate and sustain psoriasis. The differentiated THP1 cells, stimulated by imiquimod (IMQ), were utilized as the activated macrophage model. IMQ was also employed to produce psoriasis-like lesions in mice. A transcriptomic assay of macrophages revealed that the expressions of pro-inflammatory mediators and GDAP1L1 were largely increased after an IMQ intervention. The depletion of GDAP1L1 by short hairpin (sh)RNA could inhibit cytokine release by macrophages. GDAP1L1 modulated cytokine production by activating the phosphorylation of mitogen-activated protein kinases (MAPKs) and nuclear factor (NF)-κB pathways. Besides GDAP1L1, another mitochondrial fission factor, Drp1, translocated from the cytosol to mitochondria after IMQ stimulation, followed by the mitochondrial fragmentation according to the immunofluorescence imaging. Clodronate liposomes were injected into the mice to deplete native macrophages for examining the latter’s capacity on IMQ-induced inflammation. The THP1 cells, with or without GDAP1L1 silencing, were then transplanted into the mice to monitor the deposition of macrophages. We found a significant THP1 accumulation in the skin and lymph nodes. The silencing of GDAP1L1 in IMQ-treated animals reduced the psoriasiform severity score from 8 to 2. After depleting GDAP1L1, the THP1 recruitment in the lymph nodes was decreased by 3-fold. The skin histology showed that the GDAP1L1-mediated macrophage activation induced neutrophil chemotaxis and keratinocyte hyperproliferation. Thus, mitochondrial fission can be a target for fighting against psoriatic inflammation.

## 1. Introduction

One of the most widespread autoimmune cutaneous disorders is psoriasis. This disease affects 0.5–11% of the global population, depending on geographic distribution, age, and ethnicity [1]. Psoriasis is clinically diagnosed by silver scales, squamous erythema, and a thickened epidermal layer. At the cellular level, the interplay between immune cell infiltration and keratinocyte overproliferation is involved in the psoriatic pathogenesis to induce the vicious cycle of inflammation [2]. Clinicians face some challenges from psoriasis, such as high prevalence, disfiguration, disability, and comorbidity. The associated comorbid diseases include cardiovascular disorders, metabolic syndromes, lymphoma, and mental illness [3]. In general, psoriasis is considered a disease predominantly mediated by T cells and dendritic cells [4]. CD3^+^ T lymphocytes and CD11c^+^ dendritic cells are reported to play a vital role in psoriatic pathogenesis [5]. In addition, there is increasing evidence showing the key role of macrophages in the development of psoriatic lesion [6]. Macrophages are largely found to release cytokines and chemokines in the early lesional site of psoriasis [7]. Therefore, regulation of macrophage recruitment and polarization can be an effective target for psoriasis management.

Although the effect of macrophages on psoriasis development is confirmed, the underlying mechanisms of macrophages in this process are not fully elucidated to this day. Therefore, it is essential to understand better the molecular pathways of macrophages for identifying an efficient therapeutic approach to control the psoriasis progression. Our purpose is to explore the possible psoriasis-related pathways in macrophages in the present study. We first employed a transcriptomic assay to find the important biomarkers related to the activated macrophages after imiquimod (IMQ) stimulation. IMQ is a toll-like receptor (TLR)7/8 ligand to trigger psoriasiform lesions in mice. This psoriasis-like plaque is initiated by the T helper (Th)17 axis with similar characteristics of human psoriasis [8]. The TLR7 stimulation can polarize macrophages to produce pro-inflammatory cytokines, leading to cutaneous inflammation [9]. Our transcriptomic analysis evaluated by microarray showed a large upregulation of cytokines and chemokines after IMQ intervention on macrophages. Besides the pro-inflammatory mediators, ganglioside-induced differentiation-associated protein 1 like 1 (GDAP1L1) is the marker exhibiting a significant level of increase in activated macrophages.

Owing to its central role in mitochondrial fission, GDAP1L1 is a homologue of GDAP1 implicated in cellular processes, such as cell death, autophagy, aging, and immune response regulation [10]. The balance between fission and fusion is critical to maintaining mitochondrial function and biogenesis. The enhanced fission and decreased fusion occur under the condition of high-level stress [11]. Mitochondrial fission represents a major cause that elicits inflammation through the control of pro-inflammatory mediators [12]. The impact of mitochondrial fission and GDAP1L1 on human diseases is extensively studied for neurodegenerative and cardiovascular disorders [13]. However, there are very few investigations about the role of fission or GDAP1L1 on psoriasis. The mitochondrial fission always needs the dynamin-related protein (Drp)1 translocation from cytoplasm to mitochondria [14]. We aimed to explore the possible molecular signaling of the GDAP1L1/Drp1 axis involved in activated macrophages in psoriasis. Both cell- and animal-based studies were performed in this work by using IMQ as the stimulator. The short hairpin (sh)RNA was used to silence the GDAP1L1 gene for verifying the role of this fission factor on psoriasiform inflammation. Since the topical IMQ can evoke the systemic inflammatory response occurring at a distance in lymph nodes and spleen^8^, the capacity of macrophages in both local skin and systemic circulation on psoriasiform inflammation was examined.

## 2. Results

### 2.1. Transcriptomic Analysis Reveals the Significant Elevation of GDAP1L1 in IMQ-Stimulated Macrophages

**Figure 1:**
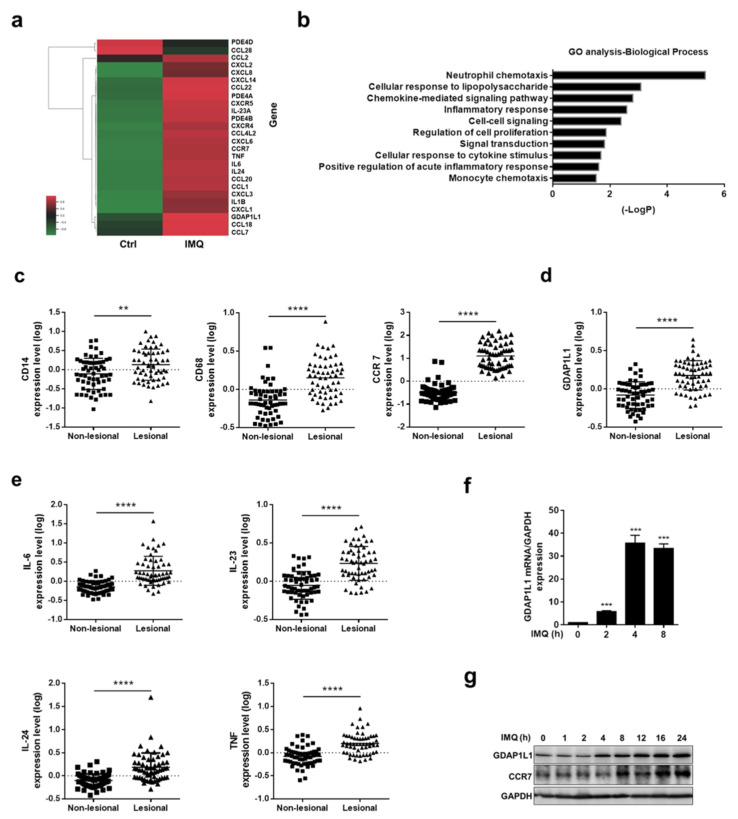
GDAP1L1 and inflammatory cytokines were upregulated in IMQ-induced macrophages and psoriatic patients. (**a**) Selected immune-associated genes significantly increased and (**b**) GO cluster classified the differentially expressed genes in macrophages by IMQ stimulation (fold ≥ 1.5). Gene expression data of skin from psoriatic patients in Gene Expression Omnibus (GEO) dataset GSE13355 were analyzed for (**c**) the expression of the macrophage markers CD14, CD68, and CCR7, and (**d**) the mitochondrial fission factor GDAP1L1, as well as (**e**) the expression of the key inflammation cytokines IL-6, IL-23, IL-24 and TNF in tissue with and without lesions from psoriatic patients (*n* = 58). (**f**,**g**) q-PCR analysis (*n* = 3) and immunoblotting analysis of the GDAP1L1 in IMQ-stimulated macrophages at the indicated periods. ** *p* < 0.01, *** *p* < 0.001, and **** *p* < 0.0001 when compared to the control (non-lesional). The data are presented as the mean ± SEM and are representative of at least three independent experiments.

Transcriptomic profiling by combined RNA sequencing and microarray is beneficial for unveiling the regulatory network of underlying diseases. Furthermore, the transcriptome was used to reveal the differentially expressed coding genes (DEGs) by comparing the macrophages with and without IMQ activation. A total of 4938 DEGs were identified between the untreated and IMQ-treated groups. We selected the DEGs commonly involved in psoriatic pathogenesis for showing the heatmap (Figure 1a). These included cytokines, chemokines, and phosphodiesterases. The heatmap of DEGs displayed global transcriptome change after receiving IMQ. In particular, the upper part of the heatmap shows 2 genes having a significant negative fold-change while 24 DEGs have a positive fold-change as shown at the lower part. CXCL2 was the DEG exhibiting the greatest increase in the stimulated THP1 (189 folds), followed by CCR7 (164 folds), CXCL3 (148 folds), and CXCL1 (117 folds). All of these genes were categorized as chemokines and the corresponding receptors. The RNA sequencing data also suggested a high upregulation of GDAP1L1 by 84-fold in IMQ-treated cells compared to the untreated group. The functional gene enrichment and annotation were then analyzed with GO to summarize the pathway network’s biological function. The neutrophil-related chemotaxis was the most significantly enriched function cluster for IMQ-activated macrophages (Figure 1b). DEGs were also enriched in the clusters concerning immune response, including cellular response to lipopolysaccharide, chemokine-mediated signaling, inflammatory response, and cellular response to cytokine.

The transcriptomic assay for psoriasis has been evaluated using large patient cohorts for comparing the gene expression between normal and lesional sites. We employed the gene set deposited by Nair et al. (GSE13355) to correlate with the transcriptome detected in macrophages. GSE13355 was extracted from the GEO databank. We first estimated the expression of macrophage markers in lesional skin to investigate the role of macrophages in psoriatic inflammation. Both CD14 and CD68 are pan-macrophage markers, while CCR7 is a hallmark of M1 macrophages. A significantly greater increase of these macrophage-associated markers was detected in the lesional than in the normal skin (Figure 1c). We also investigated whether GDAP1L1 demonstrated an increase in lesional skin as compared to non-lesional skin. Similar to the result of IMQ-stimulated THP1, GDAP1L1 in lesional skin was significantly higher (*p* < 0.0001) than that in the non-lesional area (Figure 1d). The expression of macrophage-related cytokines such as IL-6, IL-23, IL-24, and TNF in GSE13355 was analyzed. Furthermore, the results indicated a significantly enhanced expression of these cytokines in a psoriatic tissue than normal skin (Figure 1e), suggesting associated cytokines and inflammation in the psoriatic lesion.

To further elucidate the role of GDAP1L1 on IMQ-activated macrophages, a quantitative RT-PCR was conducted to detect GDAP1L1 mRNA level for the indicated time (Figure 1f) (maximum of 4 h). Furthermore, protein level was used to verify the expression of GDAP1L1 in macrophages. Meanwhile, the ELISA result showed consistency with the RT-PCR (Figure 1g and Figure S1a). It must be noted that the protein level of GDAP1L1 was respectively increased with IMQ treatment duration, a trend also observable in the case of the cell surface marker of macrophages (CCR7). We found a possible capacity of GDAP1L1 in the activation of macrophages by IMQ.

### 2.2. GDAP1L1 Is Critical in Regulating Mitogen-Activated Protein Kinases (MAPKs) and Cytokines in IMQ-Stimulated Macrophages

**Figure 2:**
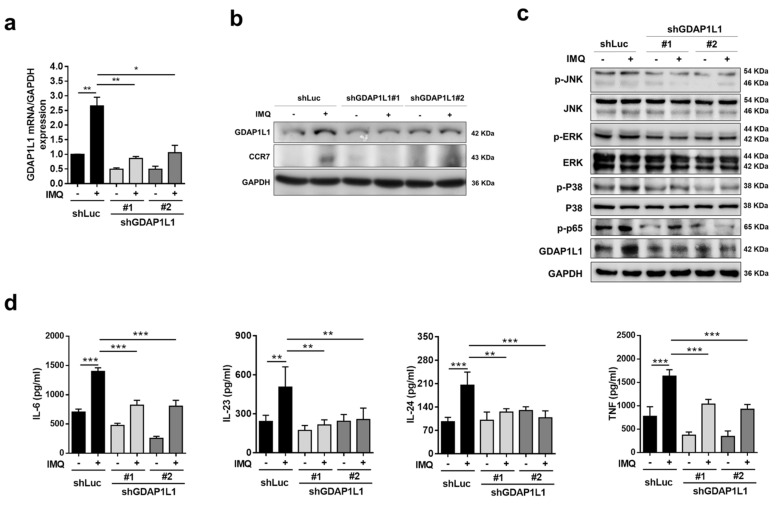
Loss of GDAP1L1 reduced IMQ-induced cytokine production and MAPK and NF-κB phosphorylation. (**a**) q-PCR analysis (*n* = 3) of GDAP1L1 and (**b**) immunoblotting analysis of the GDAP1L1 and CCR7 in control (shLuc) or GDAP1L1-deficient THP1 macrophages by IMQ stimulation. (**c**) Immunoblotting analysis of MAPKs and p65 phosphorylation levels in the control or GDAP1L1-deficient THP1 macrophages by IMQ stimulation for 4 h. (**d**) ELISA analysis of the indicated cytokines (*n* = 4−6) in the supernatants of control or GDAP1L1-deficient THP1 macrophages stimulated with IMQ for 24 h. * *p* < 0.05, ** *p* < 0.01, and *** *p* < 0.001 when compared to the control (shLuc). The data are presented as the mean ± SEM and are representative of at least three independent experiments.

To appraise the molecular mechanism by which GDAP1L1 regulated the macrophage activation by IMQ, we depleted this fission factor in THP1 cells by two lentivirus-mediated shRNAs (shGDAP1L1#1 and #2). A control shRNA (shLuc) was also used in this study. The mRNA level of GDAP1L1 was increased in shLuc-expressed macrophages stimulated with IMQ, whereas the treatment of shGDAP1L1 on THP1 resulted in a significant depletion of GDAP1L1 (Figure 2a). The shGDAP1L1#1 and #2 markedly reduced GDAP1L1 in activated THP1 by 80% and 60%, respectively. The shGDAP1L1 also blocked the protein expression of IMQ-stimulated GDAP1L1 and CCR7 (Figure 2b). The signaling system of MAPKs has emerged as a target of the pathogenic pathway for psoriasis development. To test whether GDAP1L1 is involved in regulating the MAPK pathway, we determined the expression of total and phosphorylated forms of JNK, ERK, and p38 in IMQ-treated macrophages with Western blotting assay. The phosphorylated MAPKs increased after treatment with IMQ in shLuc-expressed cells (Figure 2c). The depletion of GDAP1L1 by shRNA could significantly reverse this increase by 30−70% (Figure S1b), suggesting GDAP1L1′s capability to regulate MAPK signaling. Notably, NF-κB is a downstream target of MAPKs. The shGDAP1L1 attenuated IMQ-induced phosphorylation of NF-κB p65 (Figure 2c).

The cytokines IL-6, IL-23, IL-24, and TNF are signatures of M1 macrophages. The transcriptomic analysis in psoriatic patients showed an overexpression of these cytokines in lesional tissue (Figure 1e). An ELISA test was carried out to validate the clinical result on cytokines in macrophages. Consistent with the RNA sequencing data of psoriatic patients, the protein expression of these cytokines was upregulated by IMQ treatment in shLuc-expressed THP1 (Figure 2d). The IMQ treatment promoted the cytokine expression by about 2-fold, unlike the untreated control. The silencing of GDAP1L1 inhibited the expression of pro-inflammatory mediators with statistical significance.

### 2.3. IMQ Impels the Translocation of GDAP1L1 and Drp1 from the Cytosol to Mitochondria

**Figure 3:**
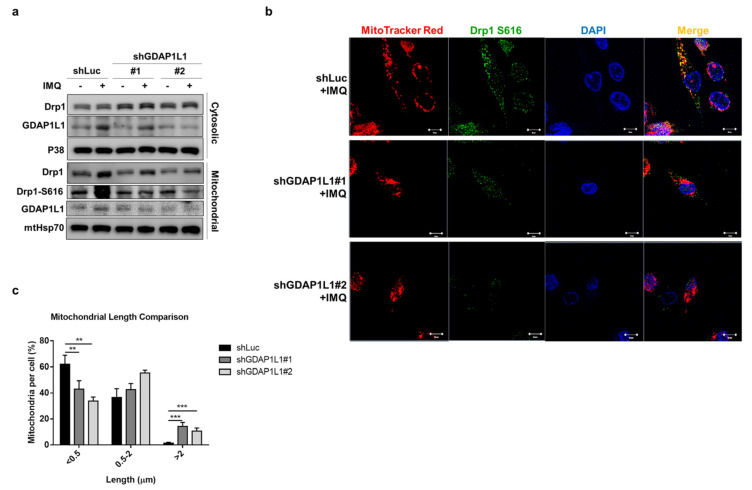
Loss of GDAP1L1 ameliorated Drp1 phosphorylation and mitochondrial translocation. Control and GDAP1L1-deficient THP1 macrophages were stimulated with IMQ (10 μg/mL) for 2 h. (**a**) Cells were fractionated into cytosolic and mitochondrial fractions and subjected to immunoblotting using the indicated antibodies. (**b**) Cells were stained with MitoTracker Red and fixed and immunostained with antibodies against GDAP1L1 and Drp1 S616, followed by confocal microscopy. Nuclei were stained blue using DAPI. (**c**) Average mitochondrial length (μm) and comparison of mitochondrial length distribution in the indicated groups. Scale bars, 10 μm. All experiments were repeated two or three times with similar results. Significant differences are indicated by ** *p* < 0.01 and, *** *p* < 0.001. Data are represented as mean ± SEM.

The induction of mitochondrial fission by GDAP1L1 depends upon the association with another fission factor, Drp1. GDAP1L1 and Drp1 are cytosolic proteins delivered from the cytoplasm to the mitochondrial surface in response to stimuli. We sought to investigate the translocation of fission factors in macrophages stimulated by IMQ. The cytosolic and mitochondrial fractions were isolated from macrophages to detect the expression of fission factors. Furthermore, the expression of both cytosolic and mitochondrial GDAP1L1 was remarkably increased in IMQ-treated THP1 expressing shLuc (Figure 3a), indicating the upregulation of total GDAP1L1 by activating macrophages. Thus, silencing GDAP1L1 could abolish this increase in both cytosol and mitochondria. Meanwhile, Drp1 was increased by IMQ treatment on shLuc-expressed macrophage mitochondria, except on cytosol. However, the GDAP1L1 depletion suppressed the translocation of Drp1 into mitochondria. In addition to Drp1 translocation, mitochondrial fission necessitates elevated phosphorylation at serine residue 616. We then examined Drp1 S616 in mitochondria, treated with IMQ. Mitochondrial Drp1 S616 was significantly increased after the IMQ intervention, while its increase was notably blocked by shGDAP1L1. The S616 protein level was about 2-fold lower in IMQ-treated GDAP1L1-silenced macrophages than in the shLuc control (Figure S1c).

We next visualized the subcellular location of Drp1 S616 using immunofluorescence staining. Mito Tracker and DAPI labeled mitochondria and nuclei to observe the localization of these organelles, respectively. Punctate and short mitochondria were visible in shLuc-expressed macrophages treated with IMQ (Figure 3b). An appreciable overlap between mitochondria and Drp1 S616 was also found. GDAP1L1 depletion converted the fragmented mitochondria to an elongated morphology. The colocalization of Drp1 S616 with mitochondria was limited when shGDAP1L1 intervened. The calculation of mitochondrial length revealed an increased population of short mitochondria after IMQ treatment on macrophages expressing shLuc (Figure 3c), demonstrating the mitochondrial dynamics of fission by IMQ. Depletion of GDAP1L1 led to mitochondrial fusion because of the formation of a tubular mitochondrial network as measured by an increased proportion of mitochondria with higher length (>2 μm). Our data suggested the possibility that IMQ elicited the phosphorylation of Drp1 and its translocation from the cytosol to mitochondria, contributing to mitochondrial fragmentation.

### 2.4. GDAP1L1 Plays a Major Role to Induce Psoriasiform Inflammation in Mice

**Figure 4:**
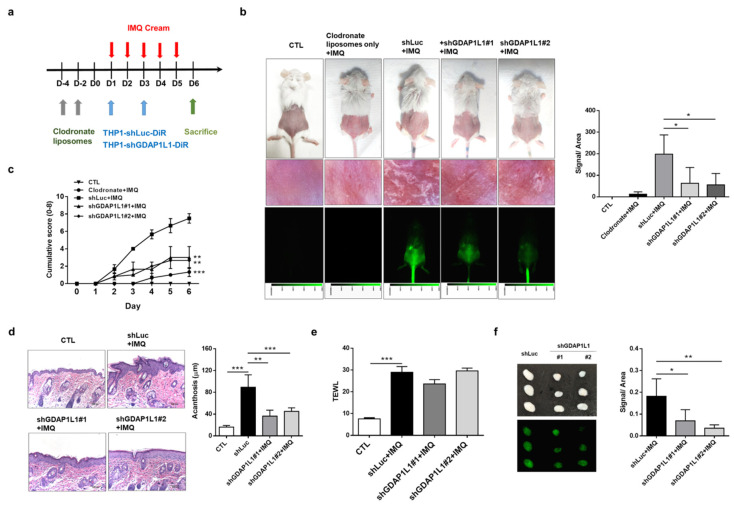
GDAP1L1 inversely regulated the macrophage numbers in lesional skin and the skin draining lymph node, and the histological changes in IMQ-induced psoriasis-like inflammation. (**a**) Experimental schedule for DiR-labeled control or GDAP1L1-deficient THP1 macrophage transplantation into the macrophage-depleted psoriasis-like inflammation model. (**b**) Phenotypical and microscopic images of IMQ-induced macrophage-depleted psoriasis-like inflammation on mouse back skin with and without control or GDAP1L1-deficient THP1 macrophage transplantation. The in vivo biodistribution of DiR-labeled THP1 cells of the skin was monitored using Pearl Impulse (Li-Cor, NE, USA) after intravenous injection in a mouse. In vivo accumulation signal in a mouse, skin injection of DiR-labeled THP1 cells, was quantified in the right panel (*n* = 6). (**c**) The cumulative score (erythema plus scaling on a scale from 0 to 4 of each) was depicted (0–8) (*n* = 6). (**d**) Histological assessment of back skin by H&E staining and thickness (μm) measured using an image analysis system in the right panel (*n* = 8). (**e**) Transepidermal water loss (TEWL) was measured at day 6 (*n* = 6). (**f**) At day 6, the lymph node was removed and visualized as shown, and ex vivo near-infrared signal of the organs was quantified (*n* = 5). All experiments were performed at least three times. * *p* < 0.05, ** *p* < 0.01 and *** *p* < 0.001 when compared to control group. Data are represented as mean ± SEM. Scale bar, 100 μm.

In an attempt to identify whether activated macrophages and GDAP1L1/Drp1 axis are mandatory in maintaining psoriasiform inflammation, we eliminated macrophages from Balb/c mice by intraperitoneal clodronate liposomes. Then, the DiR-labeled THP1 cells expressing either shLuc or shGDAP1L1 were intravenously injected into the mice to replace the native macrophages necessary in tracking the location of macrophages in psoriasis-like mice (Figure 4a). At first, we should confirm the effect of clodronate liposomes on macrophage depletion in the IMQ-stimulated mouse model. Therefore, we injected PBS liposomes as the control for comparison. Rather than control liposomes, clodronate liposome delivery eliminated scaling and erythema in IMQ-applied skin (Figure S2a). Flow cytometric analysis manifested a 70% reduction in the total F4/80 monocytes/macrophages in the blood (Figure S2b). However, this reduction was not detected in the presence of PBS liposomes. This result suggested the success of macrophage depletion by clodronate liposomes. To further investigate the effect of the homing ability of macrophages in psoriasiform inflammation, we transferred DiR-labeled THP1 cells into macrophage-depleted mice. The near-infrared imaging of the excised IMQ-treated skin illustrated a significant green signal produced by transferring DiR-labeled THP1 (Figure S3a). However, this phenomenon was absent among healthy mice and without IMQ activation. Although we observed a significant DiR intensity in the skin area with IMQ administration, this was weaker in the untreated region adjacent to the IMQ-treated area (Figure S3b). This result demonstrated that the transplantation of THP1 to macrophage-depleted mice led to a migration to psoriasiform plaque. The recruitment of DiR-labeled THP1 was visualized in the dermis of IMQ-treated mice according to the immunofluorescence histology (Figure S3c). The THP1 cells were mainly located near the vessel, demonstrating the infiltration of THP1 cells from circulation.

The macrophage-depleted animals treated with shLuc-expressed THP1 displayed typical symptoms of psoriasis, including scaling and erythema (the upper left panel of Figure 4b). Administration of shGDAP1L1-expressed THP1 in mice remarkably alleviated psoriasis-like symptoms, suggesting the connection of GDAP1L1 in lesional development. The in vivo near-infrared imaging of the skin showed a significant accumulation of shLuc-expressed THP1 on the IMQ-applied region. This deposition was minor in the shGDAP1L1-treated groups (lower-left panel of Figure 4b). The liposome-treated mice without THP1 cell transplantation exhibited minimal psoriasis-like symptoms, indicating that the macrophage depletion by clodronate liposomes could mitigate the IMQ-induced inflammation. The vital role of macrophages on the initiation of psoriasiform inflammation was thus verified. It is expected that no fluorescence signal was visualized in the skin of liposome-treated animals without THP1 cell transplantation (lower-left panel of Figure 4b). The near-infrared signal in the shGDAP1L1-treated mice was approximately 3-fold lesser than that of shLuc-treated mice (right panel of Figure 4b). The cumulative score of scaling and erythema increased with the application of IMQ on shLuc-treated mice, whereas the transplantation with shGDAP1L1-expressed THP1 significantly restrained the severity score from days 2 to 6 (Figure 4c). Histopathological observation of psoriasis-like plaque, induced by IMQ in shLuc-intervened animals, exhibited the typical hallmarks of acanthosis, parakeratosis, and immune cell accumulation (left panel of Figure 4d). The skin of IMQ-treated mice showed a 4-fold increase in epidermal thickness compared to that of healthy mice (right panel of Figure 4d). While the application of shGDAP1L1 significantly reduced epidermal acanthosis, it did not achieve a complete relief. TEWL can evaluate the skin barrier property. A 3-fold increase of TEWL was detected after IMQ treatment on shLuc-expressed animals compared to healthy control (Figure 4e). Nevertheless, silencing GDAP1L1 did not recover the barrier dysfunction caused by IMQ, suggesting GDAP1L1′s failure to govern the skin barrier property. No animal was dead after the transplantation of THP1 cells into the circulation. The vitality of these animals could be maintained during the experiments. This suggested the tolerance of the mice to transplant rejection.

The immune-mediated response of lymph nodes can be related to psoriatic severity. Since topically applied IMQ can induce the systemic immune reaction in lymph nodes, we investigated the impact of macrophages and GDAP1L1 on a skin-draining lymph node. The lymph node was dissected from the IMQ-treated mice with shLuc expression. An increase in the mass was observed by IMQ activation (upper left panel of Figure 4f). The IMQ-stimulated lymph node swelling was inhibited in the absence of GDAP1L1. The transferred DiR-labeled cells were lesser in the shGDAP1L1-treated group than the shLuc control group (lower-left panel of Figure 4f). The near-infrared intensity revealed a 3-fold decrease in the lymph node with transferred shGDAP1L1-treated THP1 compared to the shLuc group. This finding demonstrates the sufficiency of GDAP1L1 silencing in suppressing systemic macrophage migration to lymph nodes.

**Figure 5:**
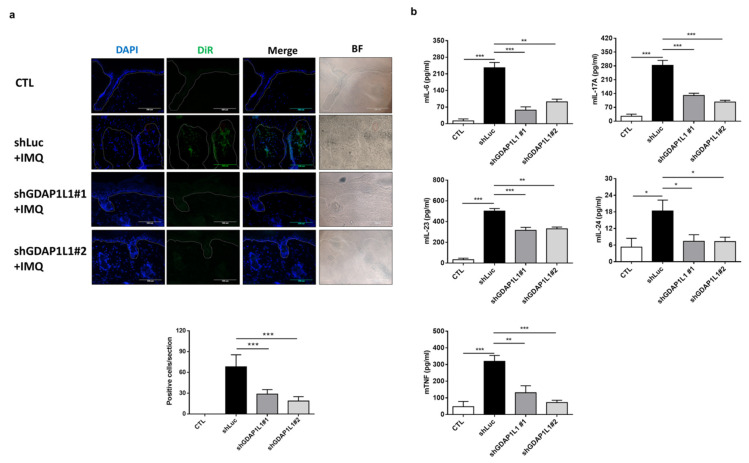
Loss of GDAP1L1 attenuated inflammation cytokine expression in an IMQ-induced mouse model. (**a**) Skin cryosections from the DiR-labeled control or GDAP1L1-deficient THP1 macrophage transplantation mice. DiR-labeled infiltrated macrophages (green) were found in the dermis and nearby thickened epidermis, as indicated by arrows. Cell nuclei (blue) were counterstained with DAPI. The dotted lines indicate the border between the epidermis and dermis. Scale bar, 100 μm. Quantitative analyses of DiR-labeled infiltrated macrophages in the skin were counted in high-power fields. (*n* = 5) is presented in the bottom panel. (**b**) Production of cytokines as indicated in the skin (*n* = 5−6) was measured with ELISA. * *p* < 0.05, ** *p* < 0.01 and *** *p* < 0.001 when compared to control group. Data are represented as mean ± SEM of three independent experiments.

We next examined the change of IMQ-stimulated skin by transplanting shGDAP1L1-treated macrophages at cellular and molecular levels. The THP1 infiltration in the skin was validated by immunofluorescence staining (upper panel of Figure 5a). The dotted line in this figure presented the dermal–epidermal junction. An abundant cluster of shLuc-expressed THP1 was accumulated in the dermis after IMQ treatment. On the other hand, some THP1 could be found in the epidermis. We could find the vessel near the dermal-epidermal junction in the shLuc-treated skin (the red circle in Figure 5a). The DiR-labeled THP1 cells were observed near the vessel, indicating the possible migration of THP1 from circulation to lesional skin tissue. Meanwhile, the DiR-labeled cells in IMQ-treated skin were limited after GDAP1L1 silencing. A 3- to 4-fold decrease of DiR-labeled THP1 could be counted after GDAP1L1 depletion (lower panel of Figure 5a). This result clearly showed the ability of GDAP1L1 to control macrophage trafficking to psoriasiform skin. Furthermore, the ELISA data showed that the pro-inflammatory mediators including IL-6, IL-17A, IL-23, IL-24, and TNF dramatically increased in psoriasis-like mice intervened by shLuc (Figure 5b). On the other hand, this increase was reduced in the mice transferred with shGDAP1L1-expressed THP1.

**Figure 6:**
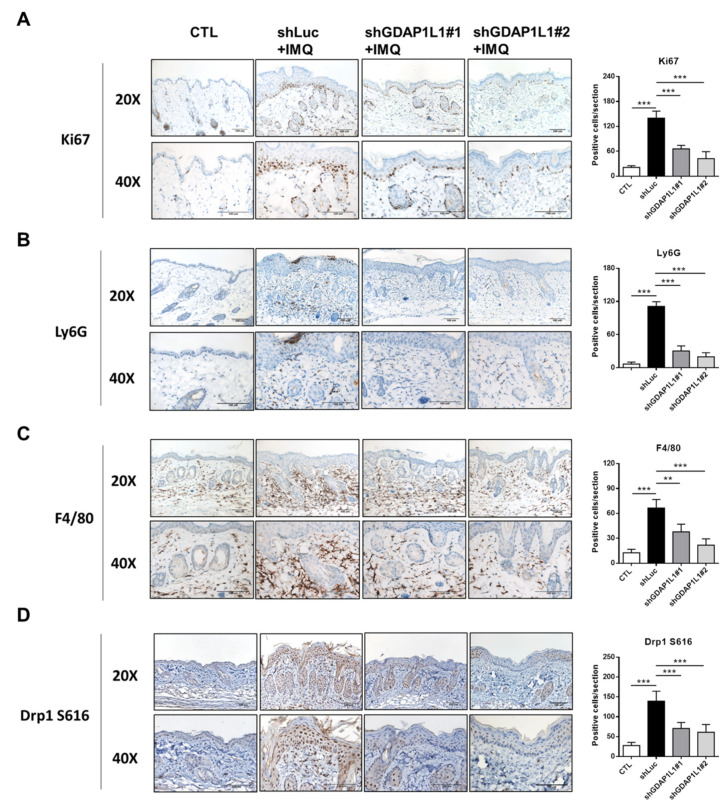
Loss of GDAP1L1 ameliorated histological changes of IMQ-induced psoriasis-like inflammation. Immunohistochemical detection of (**A**) Ki67, (**B**) Ly6G, (**C**) F4/80, and (**D**) Drp1 S616 expression in dorsal skin from the control or GDAP1L1-deficient THP1 macrophage transplantation mice with or without IMQ on Day 6. Quantitative analysis of the indicated markers in the skin (*n* = 5) is presented in the right panel. ** *p* < 0.01 and *** *p* < 0.001 when compared to shLuc group. Data are represented as mean ± SEM. Scale bar, 100 μm.

In order to survey the keratinocyte proliferation and immune cell infiltration in IMQ-activated skin, IHC analysis was performed. The representative staining of Ki-67, the proliferative marker of keratinocytes, showed increased keratinocytes in the basal layer after IMQ and shLuc treatments (left panel of Figure 6A). GDAP1L1 depletion considerably downregulated the Ki67-positive cells in IMQ-treated skin. Noteworthily, ShGDAP1L1#1 and #2 also decreased Ki67 to a similar degree (right panel of Figure 6A). IHC for Ly6G, a marker of neutrophils, in the skin from IMQ-treated shLuc-expressed mice illustrated a strong expression in the dermis (Figure 6B). The Ly6G-positive cells were also significantly accumulated in the upper epidermis (Munro’s microabscess). Furthermore, while the positive staining of Ly6G was relieved, its expression was virtually undetectable in the upper dermis in the shGDAP1L1-treated group. F4/80 is a membrane protein largely expressed on the macrophage surface. The healthy skin control exhibited some F4/80-positive cells in the dermis (left panel of Figure 6C). Meanwhile, the animals receiving IMQ and shLuc had a significantly increased level of F4/80-positive macrophages in the dermis. This increase exhibited by IMQ intervention was about 5-fold compared to the normal control skin (right panel of Figure 6C). GDAP1L1 silencing restricted the trafficking of macrophages to the dermis, especially for shGDAP1L1#2. We also monitored the F4/80 macrophage deposition in the lymph node. The same tendency in the skin tissue was found after transplanting shRNA-treated THP1 in psoriasis-like mice (Figure S4). The IMQ intervention elevated the number of infiltrating Drp1 S616-positive cells in viable skin among shLuc-treated animals (left panel of Figure 6D). The shGDAP1L1#1 and #2 treatments significantly decreased the accumulation of Drp1 S616 by 2- and 3-fold, respectively (right panel of Figure 6D). This IHC visualization confirmed the association of GDAP1L1 with Drp1 in generating psoriasiform inflammation.

## 3. Discussion

Macrophages are considered to possess a significant role in the initiation of psoriatic inflammation. In this study, the attenuation of psoriasiform mouse skin phenotype after clodronate liposomes and shRNA-treated THP1 administrations showed that macrophages were necessary for developing psoriasiform lesions. However, the mechanistic pathway of activated macrophages in the development of psoriasis is not fully explored so far. Herein, our findings demonstrated that IMQ stimulation triggered either the GDAP1L1 expression or the translocation of GDAP1L1 and Drp1 to mitochondria. We further clarified that IMQ-induced Drp1-dependent mitochondrial fission occurred in a GDAP1L1-controlled manner. Mitochondrial dynamics are important factors governing the activation of macrophages for developing psoriasiform inflammation. High-throughput evaluation of gene expression, using RNA sequencing and microarray, elucidates the disease mechanism in cell-based and clinical research [15]. Our transcriptome assay demonstrated pivotal genes of cytokines, chemokines, and GDAP1L1 associated with the molecular pathogenesis of IMQ-treated macrophages. The transcriptomic analysis based on the clinical study also confirmed the important role of macrophages and GDAP1L1 in the psoriatic skin tissue. Psoriasis is an immune-mediated inflammatory disease characterized by pro-inflammatory cell recruitment and overt keratinocyte proliferation. Our GO enrichment result exhibited cellular responses to cytokines and chemokines and cell proliferation in the biological process of macrophage activation by IMQ. All of these clusters were usually accepted components of the pathological change in psoriasis.

Macrophages are largely expressed with TLRs 7, 8, and 9 [16]. The predominant biological effect of IMQ is mediated via the agonistic activity towards TLRs 7 and 8. Psoriasis is a cytokine- and chemokine-driven disease in which the lesion is produced due to immune cell infiltration and keratinocyte hyperproliferation [17]. TLRs are the receptors capable of initiating cytokine generation. Macrophages are important players in the induction of pro-inflammatory mediators, which are thought to take part in the development of psoriatic inflammation [18]. Moreover, our data show the regulation of IL-6, IL-23, IL-24, and TNF by GDAP1L1. While macrophages are shown to have a high TNF expression [19], the activation of TLRs can trigger the production of TNFα in macrophages [20]. IL-23, having a crucial role in psoriasis initiation [21], is capable of evoking IL-6-dependent cytokine release and epidermal thickening [22]. Furthermore, both TNF-α and IL-6 can induce IL-24 production in psoriasis [23]. Macrophages are potential resources of IL-24 in mediating a direct proliferation on keratinocytes. In addition, IL-24 overexpression in psoriasis prompts the expression of pro-inflammatory chemokines CXCL1, CXCL8, and CCL20 [24]. Our transcriptomic assay confirmed the increased production of these chemokines in IMQ-activated macrophages.

The trafficking of immune cells in psoriatic tissue largely depends on the family of chemokines and their receptors. CCR7, a chemokine receptor, increases as monocytes differentiate into macrophages and subsequent to M1 polarization. TNF can associate with CCR7 to trigger psoriasis [25]. We found a reduced CCR7 after the depletion of GDAP1L1, indicating a possible involvement of GDAP1L1 in mediating CCR7-induced macrophage recruitment in psoriatic plaque. Chemokine binding to the corresponding receptors triggers the downstream signaling MAPK pathway. The TLR binding by IMQ also results in MAPK and NF-κB phosphorylation [26]. The experimental data demonstrated that GDAP1L1 regulated MAPK and NF-κB expression. Therefore, these pathways could be the downstream signaling of GDAP1L1-induced fission. ERK phosphorylation can cause the epidermal IL-24 overexpression [23,24]. Moreover, NF-κB regulates CXCL2 and CCL20 when cutaneous inflammation occurs [27]. The expression of these pro-inflammatory mediators was largely enhanced as observed in our transcriptomic profiles, indicating a vicious cycle of macrophage motivation by IMQ.

The phenotype of IMQ-treated mouse skin was similar to human psoriasis [16]. Most of the signaling pathways known to be involved in human psoriasis appear to be reflected in the IMQ-induced animal model [28]. We showed that IMQ intervention caused typical pathological and clinical features of psoriasis-like skin, as indicated by macroscopic appearance, histology, severity score, and barrier deficiency. Macrophages were primarily identified in psoriasiform plaque rather than in healthy skin. Particularly, they are differentiated from monocytes in circulation once these enter the host tissue and become affected by stimulation. IMQ activation prompted the macrophages to recruit in plaque skin. The macrophages are numerically deposited in the epidermal/dermal interface of the lesional area of psoriasis patients [29]. Our immunofluorescence histology confirmed the location of macrophages in the dermis and epidermis of psoriasis-like lesions. Moreover, the GDAP1L1 depletion remarkably suppressed macrophage infiltration and psoriasiform symptoms, supporting its crucial role in psoriasis development and maintenance. GDAP1L1, a fission factor controlling mitochondrial fission, induces fission upon translocation from the cytosol to mitochondria in response to stress condition. Imbalance of fission and fusion is reported to cause age-associated neurodegenerative illnesses, such as Parkinson’s and Alzheimer’s diseases [30]. Furthermore, the activity of GDAP1L1 is dependent on Drp1. GDAP1, a paralog of GDAP1L1, evokes mitochondrial fragmentation only in the presence of Drp1 in HeLa cells [31]. This effect might also happen in the case of macrophages.

The significant inhibition of Drp1 S616 in IMQ-induced lesions by GDAP1L1 depletion corroborated with the cell-based result of GDAP1L1 and Drp1 S616 translocation to prove the association of both fission factors. Both Drp1 translocations to mitochondria and phosphorylation at S616 are required for fission [32]. The fragmentation led to the shortened length of mitochondria, as observed in the immunofluorescence image of IMQ-treated macrophages. Mitochondrial elongation could be visualized by GDAP1L1 silencing, indicating the reversal to balanced dynamics. Few investigations have reported the relationship between Drp1 and cutaneous inflammation. Therianou et al. [33] demonstrate a significant reduction of Drp1 in lesional skin rather than in non-lesional or normal skin among psoriasis patients. On the other hand, Zhang et al. [34] report the increased expression of the Drp1 gene among atopic dermatitis patients. More studies are necessary to elucidate the possible bio functions of GDAP1L1 and Drp1 on autoimmune skin diseases. Meanwhile, we showed that GDAP1L1/Drp1 translocation prompted cytokine release in both THP1- and animal-based experiments. The previous study [35] also indicates that mitochondrial dynamic disruption leads to pro-inflammatory mediator production by microglial activation. Although our in vitro study proved the cytokine upregulation of IMQ-stimulated macrophages through GDAP1L1/Drp1 pathway, the cytokine overexpression in psoriasiform mice was more complex since many cells joined the cytokine production due to the interplay between these cells. For instance, TNF is upregulated in a broad range of cells, including macrophages, T cells, keratinocytes, and endothelial cells in psoriatic disease [36]. The positive feedback loop between immune cells and resident cells in psoriasis can further facilitate more TNF generation in lesional skin. The overexpressed TNF in psoriatic skin promotes the infiltration of circulating neutrophils to skin [37]. Our GO enrichment profiles demonstrated that neutrophil chemotaxis was the most relevant to macrophage activation by IMQ. The Ly6G-stained IHC displayed an inflammatory infiltration of neutrophils in the upper epidermis and dermis in the psoriasiform lesion. In addition to macrophages, T cells and keratinocytes can induce IL-17A production when activated [38,39]. IL-17A can act synergistically with TNF-α to elicit neutrophil accumulation in psoriatic skin [40]. Our data showed a significant IL-17A expression enhancement after IMQ treatment on the skin, verified to be related to GDAP1L1 signaling. Macrophages accumulated in lesions release cytokines to stimulate keratinocytes [18], resulting in excessive proliferation as found in our histological result. It must be noted that this hyperproliferation was initiated not only by macrophages but also by the other immunocyte subsets.

Besides the psoriatic lesional skin, the stimulated macrophages are largely found in the lymph node [41]. Although IMQ is used to induce inflammation via topical delivery, it appears to cause a systemic immunological response in the lymph node [42,43]. Our data indicated the accumulation of macrophages in the lymph node after topical IMQ application, which also apparently increased the lymph node’s mass. The IMQ-treated mice had been pretreated with clodronate liposomes to deplete native macrophages. On the other hand, the clodronate liposomes are utilized to remove macrophages by apoptosis from the liver, spleen, lymph node, and skin with no influence on T cells, neutrophils, mast cells, and Langerhans cells [44]. We showed that clodronate liposomes could deplete most of the macrophages. Nevertheless, there were still some native macrophages that remained in the mice. Thus, macrophage recruitment in the lymph node and skin could be attributed to both the remaining native macrophages and transplanted THP1. Moreover, our study discovered that GDAP1L1 controlled cutaneous inflammation induced by IMQ and the systemic immune response. The activated macrophages in circulation migrated to the skin-draining lymph and remodeled to M1 type for differentiating T lymphocytes to pathogenic Th17 cells by cytokine secretion [45]. Then, the activated T cells proliferated and infiltrated from circulation to skin for creating Th17-driven inflammation. Subsequently, a vicious cycle was established. The Th17-driven inflammation could attract the infiltration of macrophages or THP1 cells from the circulation to the lesional skin. Our data showed the cumulative THP1 cells in the IMQ-treated skin, and this deposition was reduced by GDAP1L1 depletion. The silencing of GDAP1L1 might inactivate macrophages to lose the ability of spreading. GDAP1L1 depletion retarded THP1 migration to lymph nodes, leading to the subsequent prevention of the vicious inflammation cycle and the THP1 movement to lesional skin. We anticipated an important capacity of GDAP1L1/Drp1 signaling in this positive feedback loop.

**Figure 7:**
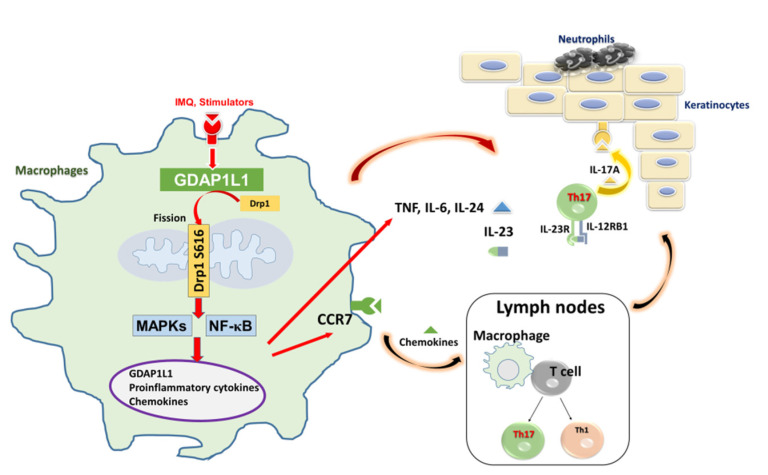
Schematic representation of GDAP1L1-modulated macrophage trafficking and inflammation cytokine responses to regulate psoriatic skin inflammation. The activation of antigen-presenting cells, such as macrophages, initiated the development of psoriatic inflammation. The ligand of TLR 7/8, IMQ, activated cytokine and chemokine production in macrophages through MAPK and NK-κB phosphorylation, which was in the GDAP1L1-dependent manner. Activated macrophages could traffic to the skin-draining lymph node and the dermis via vessel and contribute to T cell-mediated and epidermis-mediated psoriasiform skin inflammation. In turn, the cytokines and chemokines from activated macrophages rescued neutrophils, which caused epidermal proliferation. We found that IMQ induced GDAP1L1-dependent Drp1 S616 phosphorylation and then caused mitochondria translocation of GDAP1L1 and phosphorylated Drp1 S616, leading to mitochondrial fission, which played an important role in macrophage trafficking and inflammatory cytokine production.

Our result described a fundamental contribution of macrophages on the establishment of IMQ-induced psoriasiform inflammation. Mitochondrial fission, involved in GDAP1L1 and Drp1, regulated the macrophage activation by IMQ. Furthermore, our finding found that IMQ elicited GDAP1L1 expression and the translocation of Drp1 to mitochondria. The mitochondrial fission controlled the expression of pro-inflammatory mediators through MAPK and NF-κB phosphorylation in macrophages (Figure 7). Meanwhile, the stimulated macrophages could establish the crosstalk to the other immune cells and keratinocytes for creating local and systemic vicious inflammation cycle. The identification of GDAP1L1/Drp1 as the pivotal factors in psoriasis’ pathogenesis can help develop therapeutic strategies targeting mitochondrial dynamics.

Some limitations were found in the present study. Although IMQ is considered a promising approach to mimic human psoriasis for cell- and animal-based research, there are still some differences between the pathogenesis of experimental and human psoriasis. Whether the GDAP1L1/Drp1 axis is also crucial in clinical psoriasis requires further justification. Psoriasis is a very complex disease concerning the interplay of various cells. In addition to macrophages, the impact of GDAP1L1/Drp1 on the other immune or resident cells should be further investigated. Clodronate liposomes do not deplete only macrophages but also dendritic cells. Because the depletion was not specific, making it impossible to rule out other cells’ influences, some inferences on macrophages raised in this study could not be fully confirmed.

## 4. Materials and Methods

### 4.1. Materials and Antibodies

IMQ and phorbol myristate acetate (PMA) were purchased from Sigma-Aldrich (St. Louis, MO, USA). Aldara cream with 5% IMQ was a gift from 3M Pharmaceuticals (Leicestershire, UK). The antibodies of c-JUN N-terminal kinase (JNK), extracellular signal-regulated kinase (ERK), p38, C-C motif chemokine receptor (CCR)7, Drp1, and glyceraldehydes 3-phosphate dehydrogenase (GAPDH) were obtained from Santa Cruz Biotechnology (Santa Cruz, CA, USA). GDAP1L1, F4/80, and Ki67 antibodies were provided by Proteintech (Rosemont, IL, USA). Ly6G antibody was purchased from BioLegend (San Diego, CA, USA), while the mitochondrial heat shock protein 70 (mt-HSP70) antibody was purchased from Invitrogen (Carlsbad, CA, USA). Lastly, the antibody targeting Drp1-S616 was from Biorbyt (St. Louis, MO, USA).

### 4.2. Cell Culture

The THP1 cells, which are human monocyte cells, were maintained in RPMI1640 supplemented with 10% heat-treated fetal bovine serum (FBS) and 100 U/mL antibiotic-antimycotic. The cells were differentiated into macrophage-like cells by 100 ng/mL PMA treatment for 36 h, followed by overnight incubation in a fresh medium.

### 4.3. shRNA-Mediated Gene Silencing

GDAP1L1 shRNA lentivirus was produced by the National RNAi Core Facility (Taipei, Taiwan). The target sequences are summarized in Table S1. The infected cells were selected in a medium containing 2 μg/mL puromycin until the uninfected cells were completely killed. Then, the stable colonies were pooled together for further experiments.

### 4.4. Transcriptomic Analysis

Based on the manufacturer’s instruction, the total RNA from THP1 (10 μg/mL), with or without THP1 and IMQ intervention for 24 h, was collected using the Direct-zol MiniPrep RNA isolation kit. The purified RNA was quantified at OD260 nm by using a spectrophotometer (ND-1000; Nanodrop Technologies, Inc., Wilmington, DE, USA) while the integrity and concentration of total RNA samples were quantified through the RNA 6000 LabChip kit (Agilent, Santa Clara, CA, USA) and analyzed using a Bioanalyzer 2100 (Agilent). Microarray experiments were done following Welgene Biotech’s protocols. Briefly, the total RNA (0.2 μg) was amplified by a Low Input Quick-Amp Labeling kit (Agilent) and labeled with Cy3 (CyDye, Agilent Technologies, USA) during the in-vitro transcription process. A 0.6-μg Cy3-labeled cRNA was fragmented to an average size of about 50−100 nucleotides by incubation with fragmentation buffer at 60 °C for 30 min. Correspondingly fragmented labeled cRNA was then pooled and hybridized into Agilent SurePrint Microarray (Agilent) at 65 °C for 17 h. After washing and drying by nitrogen gun blowing, microarrays were scanned with an Agilent microarray scanner (Agilent) at 535 nm for Cy3. Feature extraction 10.5.1.1, image analysis, and normalization software (Agilent) analyzes scanned images to quantify signal and background intensity for each feature. Raw signal data were normalized by quantile normalization to find the differential gene expression. In addition, we provided an enrichment test for the said differential expression as a supplement to a functional assay. Furthermore, Welgene Biotech used in the enrichment rest a cluster Profiler for gene ontology (GO). The top ten enrichment GO terms obtained were then listed for pathway analysis. The datasets of transcriptomic analysis for this study can be found on the website Gene Expression Omnibus (GEO, https://www.ncbi.nlm.nih.gov/geo/, accessed date: 9 April 2021), with the access number GSE171667. As the GEO allowed us to download experiments and analyze gene expression profiles, we employed its database to search the microarray dataset of marker genes in normal and lesional tissues from psoriasis patients (GSE13355).

### 4.5. Total mRNA Extraction and Quantitative Real-Time Polymerase Chain Reaction (RT-PCR)

The total cellular RNA was extracted using a Direct-zol RNA isolation kit with RNase-free DNase 1 digestion to avoid genomic DNA contamination. Total RNA (1 μg) was reversely transcribed to cDNA using an iScript cDNA synthesis kit (Bio-Rad, Hercules, CA, USA). Quantitative PCR was carried out on a CFX Connect RT-PCR Detection System (Bio-Rad) using iQ SYBR Green Supermix. A 1/20th volume of cDNA was utilized to conduct quantitative PCR using Maxima SYBR Green/Fluorescein PCR Master Mix (Thermo, Waltham, MA, USA) based on the manufacturer’s recommendation. The primer sequences used for amplification from mice and humans are shown in Tables S1 and S2, respectively. The housekeeping GAPDH expression was used to normalize the data for gene expression.

### 4.6. Immunoblotting Assay

The THP1 cells were lysed, and the whole-cell extract was collected and quantified by a Bradford assay (Bio-Rad). The equal amounts of cell lysate were then resolved by SDS/PAGE and transferred to the PVDF membrane (Millipore, Burlington, MA, USA). The membrane was incubated with the indicated primary antibody followed by a horseradish peroxidase-conjugated secondary antibody. Meanwhile, the immunoreactive bands were detected by using Western Lighting Plus-ECL (PerkinElmer, Waltham, MA, USA). The protein level was quantified by the ratio of the densitometric measurement of the indicated proteins and the GAPDH.

### 4.7. Enzyme-Linked Immunosorbent Assay (ELISA)

The sample lysates from the supernatant of the THP1 cell culture and homogenized mouse skin were quantified by ELISA based on the manufacturer’s instruction. The level of IL-6, IL-17A, IL-23, and TNF was estimated using commercial kits (BioLegend). In addition, a Duoset ELISA kit (R&D Systems) was used to detect the IL-24.

### 4.8. Isolation of Cytoplasmic and Mitochondrial Fractions

The mitochondrial fraction was prepared as described earlier [46]. In brief, the differentiated THP1 cells of each group were collected. A Mitochondria Isolation kit (Abcam) was used to separate the cellular components, as instructed by the manufacturer. Cytoplasmic and mitochondrial proteins were quantified by Bradford assay and used for immunoblotting. Further, the levels of GDAP1L1, Drp1, and Drp1 S616 in cytoplasm and mitochondria were detected by Western blotting. The p38 and mtHSP70 were used as the loading control for the cytosolic and mitochondrial fraction, respectively.

### 4.9. Immunofluorescence Staining

The THP1, treated with or without shRNA, was differentiated and cultured on a coverslip. It was then stimulated for mitophagy induction in the presence of IMQ. After the induction of mitophagy, the mitochondria were labeled with MitoTracker Red. The cells were fixed with 4% paraformaldehyde for 15 min. The fixed cells were permeabilized with Triton X-100/PBS for 5 min and then blocked with 1% bovine serum albumin/PBS for 30 min. The immunofluorescence staining was conducted with a primary antibody against Drp S616 and a secondary antibody conjugated with Alexa Fluor 594. The THP1 cells were visualized by a confocal microscope (LSM 780, Zeiss). The measurement of mitochondrial length was conducted with MetaMorph (Molecular Devices) by Dr. Shih-Yi Chuang, blinded from the experimental groups. Moreover, the mitochondrial length was classified into three categories: <0.5 μm, 0.5−2 μm, and >2 μm.

### 4.10. Animals

Female Balb/c mice at 6−8 weeks were obtained from the National Laboratory Animal Center (Taipei, Taiwan). All experimental protocols in the present study were approved by the Institutional Animal Care and Use Committee of Chang Gung University (CGU108-101). Furthermore, all animals were housed and handled based on the Guidelines of Directive 86/109/EEC from the European Commission.

### 4.11. The Psoriasis-like Mouse Model

The mice received a daily dose of 62.5 mg IMQ in Aldara cream on the shaved area of dorsal skin for 5 days. The surface change in the dorsal region was monitored by a handheld digital magnifier (MiniScope-V, M&T Optics). The cumulative score (scaling and erythema) was estimated to exhibit the severity of skin inflammation (scale 0−8) based on the Psoriasis Area and Severity Index (PASI). Transepidermal water loss (TEWL) was recorded by TM300 Tewameter (Courage & Khazaka) each day for 6 days after the first application of the Aldara cream. The mice were sacrificed on day 6 for further examination of skin histology and cytokine expression. To deplete the macrophages in the mice, 200 μg of clodronate liposomes (Liposoma BV) was intraperitoneally administered on day 4 and 2 before IMQ application. The PBS liposomes were injected as the control. The THP1 was labeled with DiR (ThermoFisher), a near-infrared cyanine dye, with a concentration of 2 μM for 30 min. Then, the cells were washed 3 times by PBS to remove the free DiR. The DiR-labeled THP1 cells (10^6^ cells in 100 μL) were injected into mice via the intravenous route on day 0 and day 2 after IMQ treatment.

### 4.12. Histopathological Observation

The hematoxylin and eosin (H&E) and immunohistochemistry (IHC) staining was performed as described previously [47]. In brief, the skin specimen was immersed in 10% paraformaldehyde and embedded in paraffin for sectioning and H&E staining. Regarding the IHC, the skin section was treated with anti-Ki67, anti-Ly6G, anti-F4/80, or anti-Drp1 S616 antibody with a 1:100 dilution ratio. The photomicrograph was visualized under an optical microscope (DMi8, Leica). The quantification of the antibody-stained section was carried out by AlphaView software. Each sample was examined independently by two investigators, Dr. Jia-You Fang and Dr. Shih-Yi Chuang, in a blinded manner. The number of positive cells was counted for three sections of three animals per group.

### 4.13. Statistical Analysis

The data were presented as the mean and standard error of the mean (SEM). The significance of differences between the groups was assessed using Student’s *t*-test. The probability level, including 0.05, 0.01, 0.001, and 0.0001, was regarded statistically significant.

## 5. Conclusions

The experimental results in this study suggested that infiltration and activation of macrophages were necessary to establish psoriasiform inflammation. Increased GDAP1L1 expression and Drp1 translocation in macrophages after IMQ stimulation demonstrated that GDAP1L1/Drp1 acted as an enhancer of the inflammatory response initiation. Moreover, IMQ-induced mitochondrial fission was vital for pro-inflammatory mediator overexpression. The mechanistic study showed that GDAP1L1/Drp1 translocation evoked cytokine and chemokine production via MAPK and NF-κB pathways. GDAP1L1/Drp1 also modulated psoriasiform inflammation by controlling macrophage distribution in the skin and the lymph node. Neutrophils and keratinocytes are the cells possibly involved in the macrophage activation, producing the vicious inflammatory cycle. Efforts aimed at inhibiting mitochondrial fission or balancing mitochondrial dynamics can be considered in the treatment of psoriasis.

## Data Availability

The datasets of transcriptomic analysis for this study can be found on the website Gene Expression Omnibus (GEO, https://www.ncbi.nlm.nih.gov/geo/ (accessed on 9 April 2021)), with the access number GSE171667. The datasets generated and/or analyzed during the current study are available from the corresponding author on reasonable request.

## Supplementary Materials

The following are available online at https://www.mdpi.com/article/10.3390/ijms221910410/s1.
