# Supplementary material for: Psoriasiform Inflammation Is Associated with Mitochondrial Fission/GDAP1L1 Signaling in Macrophages
Source: Int J Mol Sci. 2021 Sep 27;22(19):10410. doi: 10.3390/ijms221910410 (PMC8508735; doi:10.3390/ijms221910410)
Supplement: Supplementary file 1 [file ijms-22-10410-s001.zip › ijms-1396279-supplementary.pdf]

Supplementary Table 1. shRNA target sequences used in this study

Clone ID	NM ID	Symbol	Target Sequence
TRCN0000072246	N/A	LUCIFERASE	CAAATCACAGAATCGTCGTAT
TRCN0000138924	NM_024034	GDAP1L1	GCAGACATTTAGCCAATGCCA
TRCN0000414034	NM_024034	GDAP1L1	ACGCTTTGCCTTCCGGAAAGT

Supplementary Table 2. Primer sequences used for qPCR in this study

Primer Name	Species	Forword primer sequence (5'-3')	Reverse primer sequence (5'-3')
IL-6	Mouse	TCCAGTTGCCTTCTTGGGAC	GTGTAATTAAGCCTCCGACTTG
IL-17A	Mouse	GCTCCAGAAGGCCCTCAG	CTTTCCCTCCGCATTGAC
IL-23p19	Mouse	CCCGTATCCAGTGTGAAGATG	CCCTTTGAAGATGTCAGAGTCA
IL-24	Mouse	AGAACCAGCCACCTTCACACA	CCCAAATCGGAACTCTTGACC
TNF	Mouse	CCCTCACACTCAGATCATCTTCT	GCTACGACGTGGGCTACAG
GAPDH	Mouse	AGCTTGTCATCAACGGGAAG	TTTGATGTTAGTGGGGTCTCG

Supplementary Table 3. Primer sequences used for qPCR in this study

Primer Name	Species	Forword primer sequence (5'-3')	Reverse primer sequence (5'-3')
IL-6	Human	AGCGCCTTCGGTCCAGTTGC	GTGGCTGTCTGTGTGGGGCG
IL-23p19	Human	TCCAAGCCTCAGTCCCAG	TGGGGTGGTAGATTTATCTTGG
IL-24	Human	CAGGAGGAACACGAGACTGA	GCACAACCATCTGCATTTGAGA
TNF	Human	ATGAGCACTGAAAGCATGATCC	GAGGGCTGATTAGAGAGAGGTC
GAPDH	Human	TGCACCACCAACTGCTTAGC	GGCATGGACTGTGGTCATGAG
GDAP1L1	Human	CTCTGAATGGGGCTGGATAA	ACCCAACACATGGACCAAAT

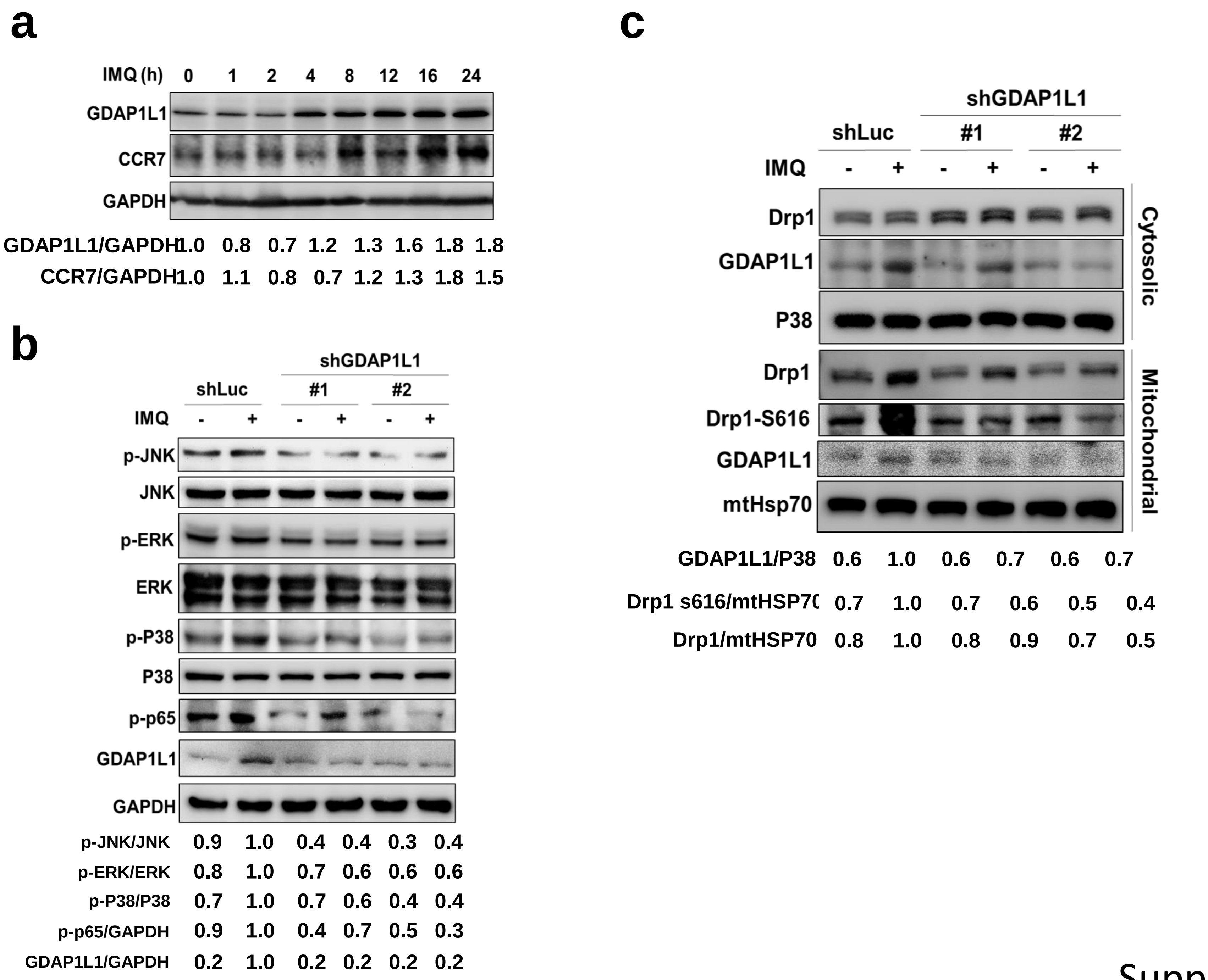
a
IMQ (h) 0 1 2 4 8 12 16 24
GDAP1L1
CCR7
GAPDH
GDAP1L1/GAPDH 1.0 0.8 0.7 1.2 1.3 1.6 1.8 1.8
CCR7/GAPDH 1.0 1.1 0.8 0.7 1.2 1.3 1.8 1.5
b
shGDAP1L1
shLuc #1 #2
IMQ - + - + - +
p-JNK
JNK
p-ERK
ERK
p-P38
P38
p-p65
GDAP1L1
GAPDH
p-JNK/JNK 0.9 1.0 0.4 0.4 0.3 0.4
p-ERK/ERK 0.8 1.0 0.7 0.6 0.6 0.6
p-P38/P38 0.7 1.0 0.7 0.6 0.4 0.4
p-p65/GAPDH 0.9 1.0 0.4 0.7 0.5 0.3
GDAP1L1/GAPDH 0.2 1.0 0.2 0.2 0.2 0.2
c
shGDAP1L1
shLuc #1 #2
IMQ - + - + - +
Drp1
GDAP1L1
P38
Cytosolic
Drp1
Drp1-S616
GDAP1L1
mtHsp70
Mitochondrial
GDAP1L1/P38 0.6 1.0 0.6 0.7 0.6 0.7
Drp1 s616/mtHSP70 0.7 1.0 0.7 0.6 0.5 0.4
Drp1/mtHSP70 0.8 1.0 0.8 0.9 0.7 0.5

Suppl. Fig. 1

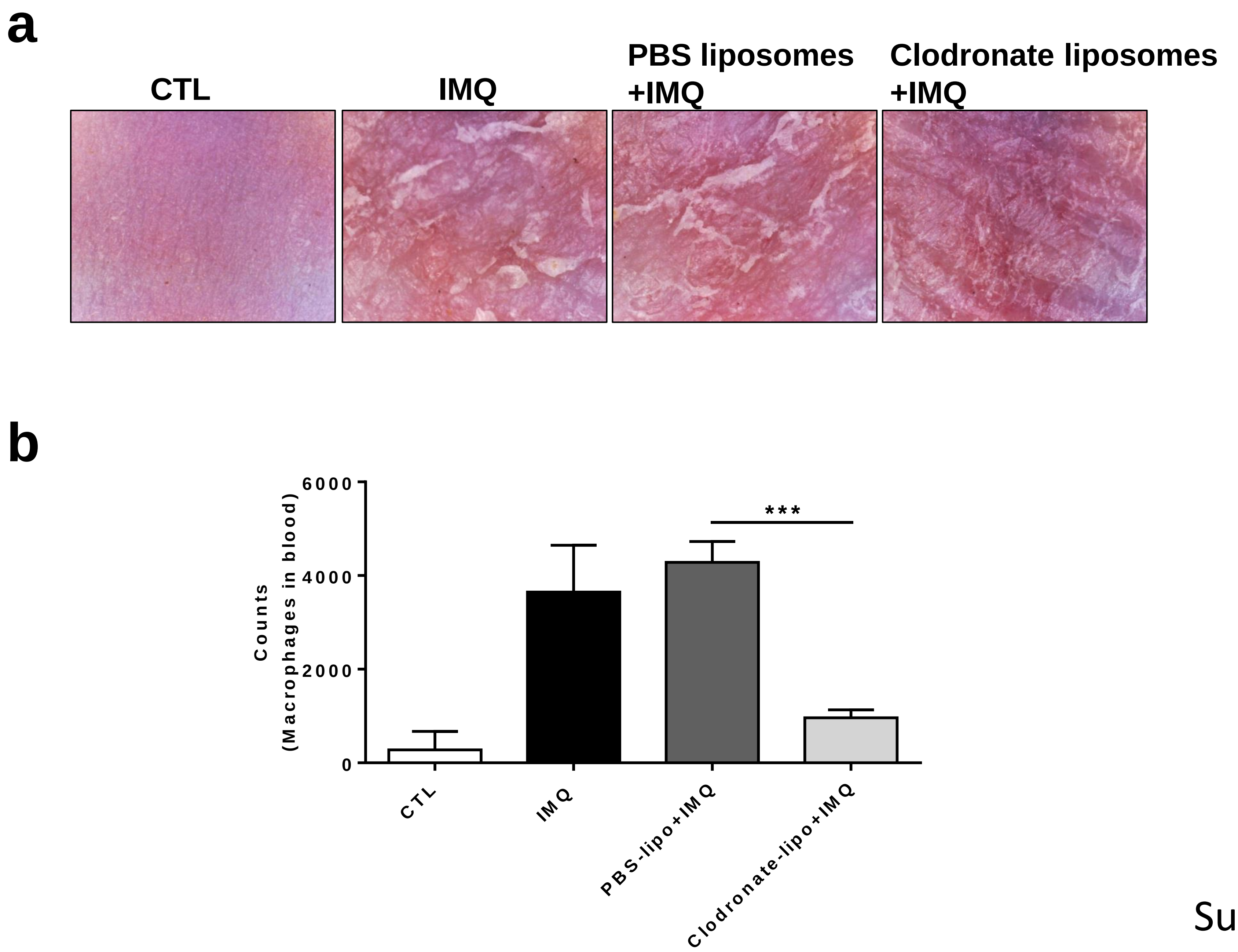

a
CTL
IMQ
PBS liposomes +IMQ
Clodronate liposomes +IMQ
b
Counts (Macrophages in blood)
6000
4000
2000
0
***
CTL
IMQ
PBS-lipo+IMQ
Clodronate-lipo+IMQ

Suppl. Fig. 2

Suppl. Fig. 3

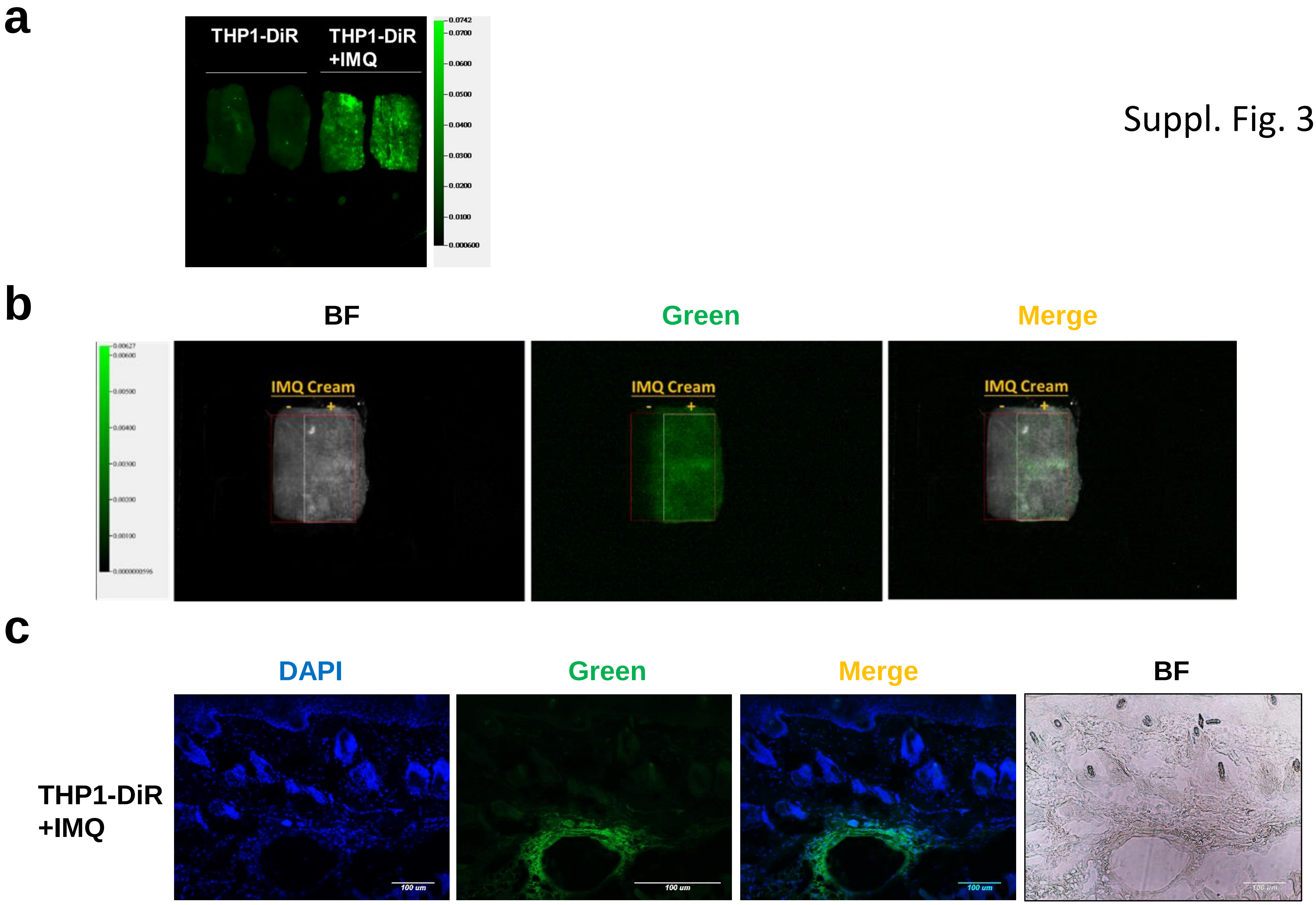
a
THP1-DiR
THP1-DiR +IMQ
0.0742
0.0700
0.0600
0.0500
0.0400
0.0300
0.0200
0.0100
0.000600
b
BF
Green
Merge
IMQ Cream
- +
c
DAPI
Green
Merge
BF
THP1-DiR +IMQ
100 um

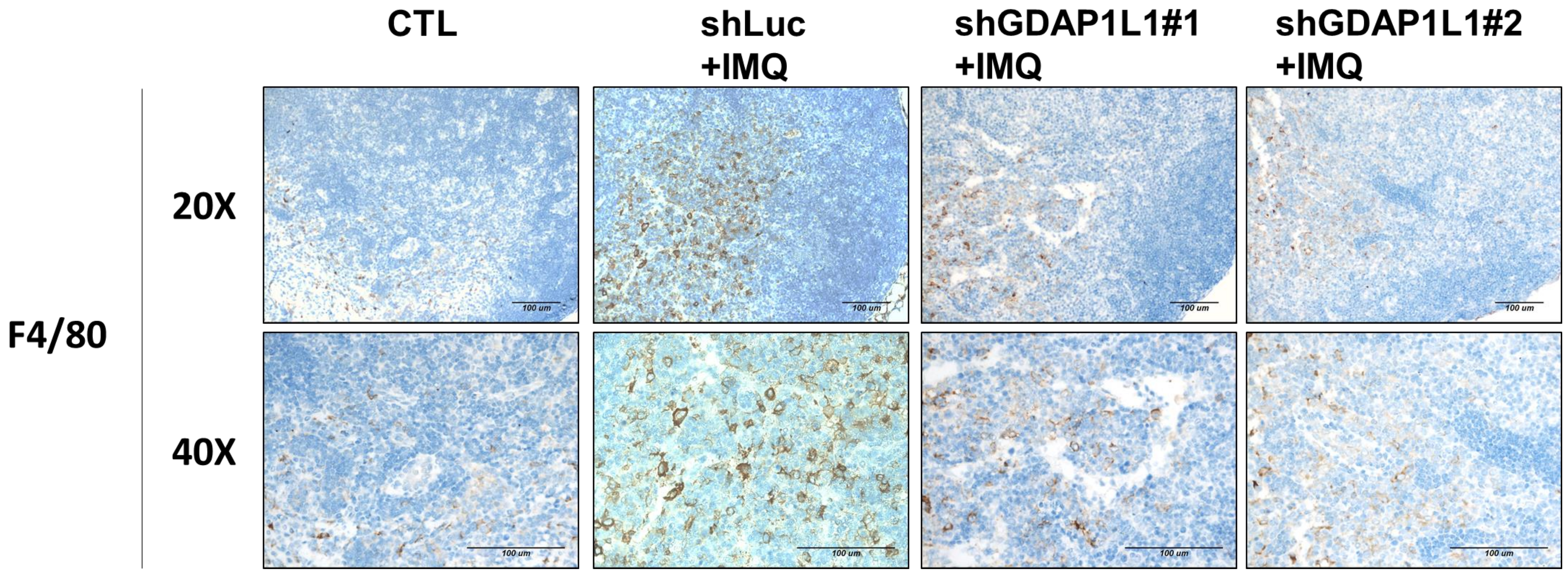
CTL
shLuc
+IMQ
shGDAP1L1#1
+IMQ
shGDAP1L1#2
+IMQ
F4/80
20X
40X
100 um

Suppl. Fig. 4

Supplementary figure legends

- Suppl. Fig. 1. The quantification of protein band by densitometry. (a) The protein/GAPDH ratio of Fig. 2b, (b) the protein/GAPDH ratio of Fig. 2c, and (c) the protein/GAPDH ratio of Fig. 3a.
- Suppl. Fig. 2. The confirmation of the effect of clodronate liposomes on macrophage depletion. (a) the skin surface image, and (b) the count of macrophages in blood.
- Suppl. Fig. 3. The distribution of injected DiR-labeled THP1 cells in skin. (a) THP1 distribution in normal and IMQ-treated mouse skin, (b) THP1 distribution in the IMQ-treated skin and the untreated skin nearby IMQ-treated region, and (c) THP1 distribution in IMQ-treated skin visualized under confocal microscopy with a vertical view.
- Suppl. Fig. 4. Macrophage accumulation in lymph node of IMQ-treated mice transplanting shRNA-treated THP1.
